# Esmethadone-HCl (REL-1017): a promising rapid antidepressant

**DOI:** 10.1007/s00406-023-01571-4

**Published:** 2023-03-08

**Authors:** Maurizio Fava, Stephen M. Stahl, Sara De Martin, Andrea Mattarei, Ezio Bettini, Stefano Comai, Andrea Alimonti, Francesco Bifari, Luca Pani, Franco Folli, Clotilde Guidetti, Alberto Furlan, Jacopo Sgrignani, Patrizia Locatelli, Andrea Cavalli, Cedric O’Gorman, Sergio Traversa, Charles E. Inturrisi, Marco Pappagallo, Paolo L. Manfredi

**Affiliations:** 1 Department of Psychiatry, Massachusetts General Hospital, Boston, MA 02114 USA; 2 Department of Psychiatry, VAMC (SD), University of California, San Diego, La Jolla, CA 92093 USA; 3 Neuroscience Education Institute, Carlsbad, CA 92008 USA; 4 Department of Pharmaceutical and Pharmacological Sciences, University of Padua, 35122 Padua, Italy; 5 In Vitro Pharmacology Department, Aptuit, an Evotec Company, 37135 Verona, Italy; 6 Department of Biomedical Sciences, University of Padua, 35122 Padua, Italy; 7 Department of Psychiatry, McGill University, Montreal, QC H3A 1A1 Canada; 8 Department of Health Sciences and Technology, ETH Zurich, 8092 Zurich, Switzerland; 9 Institute of Oncology Research (IOR), Oncology Institute of Southern Switzerland (IOSI), 6500 Bellinzona, Switzerland; 10 Università della Svizzera Italiana, 6900 Lugano, Switzerland; 11 Veneto Institute of Molecular Medicine, 35129 Padua, Italy; 12 Department of Medicine—DIMED, University of Padua, 35122 Padua, Italy; 13 Department of Medical Biotechnology and Translational Medicine, University of Milan, 20122 Milan, Italy; 14 Relmada Therapeutics, Coral Gables, FL 33134 USA; 15 Department of Psychiatry and Behavioral Sciences, School of Medicine, University of Miami, Miami, FL 33146 USA; 16 Department of Biomedical, Metabolic and Neural Sciences, University of Modena and Reggio Emilia, 41121 Modena, Italy; 17 Department of Health Sciences, University of Milan, 20122 Milan, Italy; 18 Child and Adolescent Neuropsychiatry Unit, Department of Neuroscience, Bambino Gesù Pediatric Hospital, 00165 Rome, Italy; 19 Institute for Research in Biomedicine (IRB), Università della Svizzera Italiana (USI), 6500 Bellinzona, Switzerland; 20 Swiss Institute of Bioinformatics, 1015 Lausanne, Switzerland

**Keywords:** Esmethadone, Esketamine, Ketamine, Major depressive disorder, REL-1017, N-Methyl-D-aspartate receptor

## Abstract

This review article presents select recent studies that form the basis for the development of esmethadone into a potential new drug. Esmethadone is a promising member of the pharmacological class of uncompetitive N-methyl-D-aspartate receptor (NMDAR) antagonists that have shown efficacy for major depressive disorder (MDD) and other diseases and disorders, such as Alzheimer’s dementia and pseudobulbar affect. The other drugs in the novel class of NMDAR antagonists with therapeutic uses that are discussed for comparative purposes in this review are esketamine, ketamine, dextromethorphan, and memantine. We present in silico, in vitro, in vivo, and clinical data for esmethadone and other uncompetitive NMDAR antagonists that may advance our understanding of the role of these receptors in neural plasticity in health and disease. The efficacy of NMDAR antagonists as rapid antidepressants may advance our understanding of the neurobiology of MDD and other neuropsychiatric diseases and disorders.

## Introduction

The contemporary understanding of major depressive disorder (MDD) neurobiology is progressively disengaging from the classic serotonergic hypothesis [1]. Accordingly, the risk–benefit ratio of available antidepressants, which mostly target monoaminergic neurotransmissions, has been increasingly questioned [2]. More recent hypotheses implicate impairments of neural plasticity in the pathogenesis of MDD [3–5] through the dysregulation of glutamatergic signaling via N-methyl-D-aspartate receptors (NMDARs) [6, 7]. Individuals with MDD suffer not only from depressed mood but also from cognitive deficits, and animal models of depressive-like behavior display learning deficits that have also been related to the impairment of neural plasticity [8, 9]. In the prefrontal cortex and hippocampus, impairment in neural plasticity has been associated with chronic inescapable stress and other models of depressive-like behavior [10, 11]. Interestingly, patients with MDD have also been shown to have reduced hippocampal volume [12, 13]. While MDD is still primarily considered a mood disorder, the impairment of cognition and motivation may be primary for understanding the neurobiology of this disorder. Furthermore, cognitive deficits in MDD are central in determining the prominent functional loss and disability seen in patients.

In experimental models of depressive-like behavior, reduced synaptic spine volume and impaired spinogenesis are reversed by NMDAR antagonists [14–16]. Specifically, Fogaça et al. [16] demonstrated that a single dose of esmethadone increased levels of the synaptic proteins PSD95, Synapsin 1, and GluA1 in the medial prefrontal cortex (mPFC) but not in the hippocampus. In addition, Li et al. [14] reported that ketamine produces a rapid (2-h) and sustained (72-h) increase in synaptic protein levels in the mPFC and increases levels of Synapsin 1 in whole rat hippocampus. The reversal of depressive-like behavior by uncompetitive NMDAR antagonists in experimental animal models appears to be due to the restoration of synaptic proteins through a brain-derived neurotrophic factor (BDNF)-dependent mechanism [14–16].

**Fig. 1:**
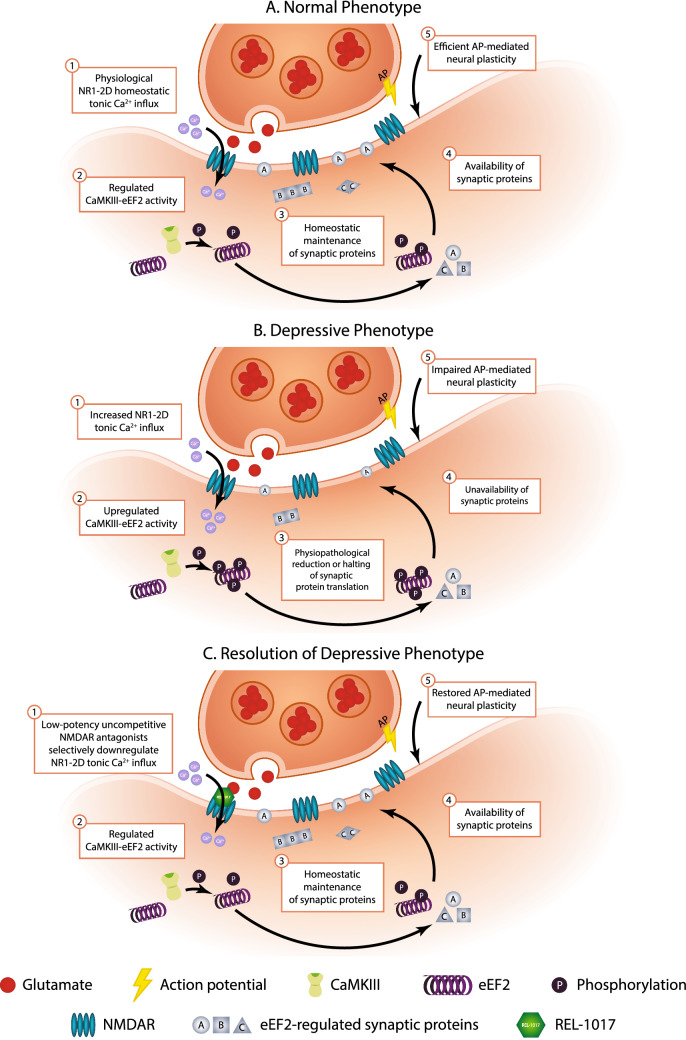
Proposed mechanism of kinase involvement in uncompetitive NMDAR antagonist-mediated rapid antidepressant effects. **A** In the normal phenotype, physiological NR1-2D homeostatic tonic Ca^2+^ influx appropriately regulates calmodulin-dependent protein kinase III (CaMKIII) phosphorylation of eukaryotic elongation factor 2 (eEF2), which results in adequate homeostatic maintenance and availability of synaptic proteins required for action potential (AP)-mediated neural plasticity. **B** In the depressive phenotype, increased Ca^2+^ influx through NR1-2D channels upregulates CaMKIII-eEF2 activity, leading to the halting of synaptic protein production and availability, impairing AP-mediated neural plasticity. **C** Resolution of the depressive phenotype is possible through the action of uncompetitive NMDAR antagonists, such as REL-1017, which block excessive tonic Ca^2+^ currents. This blockade may restore homeostatic maintenance and availability of synaptic proteins, re-enabling physiological AP-mediated synaptic plasticity

Uncompetitive NMDAR antagonists are a relatively recently described class of molecules with potential clinical applications as rapid antidepressants. One hypothesis for the mechanism of action of uncompetitive NMDAR antagonists in the treatment of depression is shown in Fig. 1 [17]. The “disinhibition hypothesis” is an alternative hypothesis that suggests that ketamine preferentially blocks NMDARs on GABAergic inhibitory interneurons, leading to a decrease of overall inhibition. This, in turn, disinhibits excitatory neurons and enhances excitatory synaptic transmission in the mPFC [18]. Other hypotheses are centered around different receptor systems, including the opioid receptor system and the sigma-1 receptor [19, 20]. While the mechanism of action of uncompetitive NMDAR antagonists for the treatment of depression needs to be further clarified and may differ among different drugs, several uncompetitive NMDAR antagonists have shown promise as antidepressant agents. The rapid antidepressant effects of ketamine have been replicated with esketamine, which has been approved for treatment-resistant depression [21]. The dextromethorphan–bupropion combination has shown efficacy for MDD in phase 2 and phase 3 trials [22, 23] and has been recently approved for the treatment of MDD. NMDAR antagonists have been FDA-approved for the treatment of other diseases and disorders. Memantine is approved for Alzheimer’s disease, and the combination drug dextromethorphan–quinidine is approved for the treatment of pseudobulbar affect. Esmethadone increased circulating BDNF levels in healthy subjects of a phase 1 clinical study [24] and improved subjective cognitive symptoms in patients with MDD in a phase 2 clinical study [25, 26]. Esmethadone (REL-1017) showed rapid, robust, and sustained antidepressant effects in a phase 2 trial conducted in patients with inadequate response to standard antidepressants [25]. Phase 3 studies are underway.

## Esmethadone (REL-1017)

Esmethadone (d-methadone; dextromethadone; REL-1017) is the opioid inactive (*S*)-enantiomer of racemic methadone and is a novel uncompetitive NMDAR antagonist [27, 28]. Esmethadone is a promising, once-daily, oral, rapid antidepressant candidate [25]. If phase 3 results reproduce the robust and sustained efficacy seen in phase 2, esmethadone could potentially become the first-in-class agent among emerging second-generation (post-ketamine), oral, uncompetitive NMDAR antagonists with rapid antidepressant effects. This work reviews the current knowledge on the pharmacology of esmethadone and its ongoing development for the treatment of MDD.

### Interactions of esmethadone with the NMDAR in silico and in vitro

**Fig. 2:**
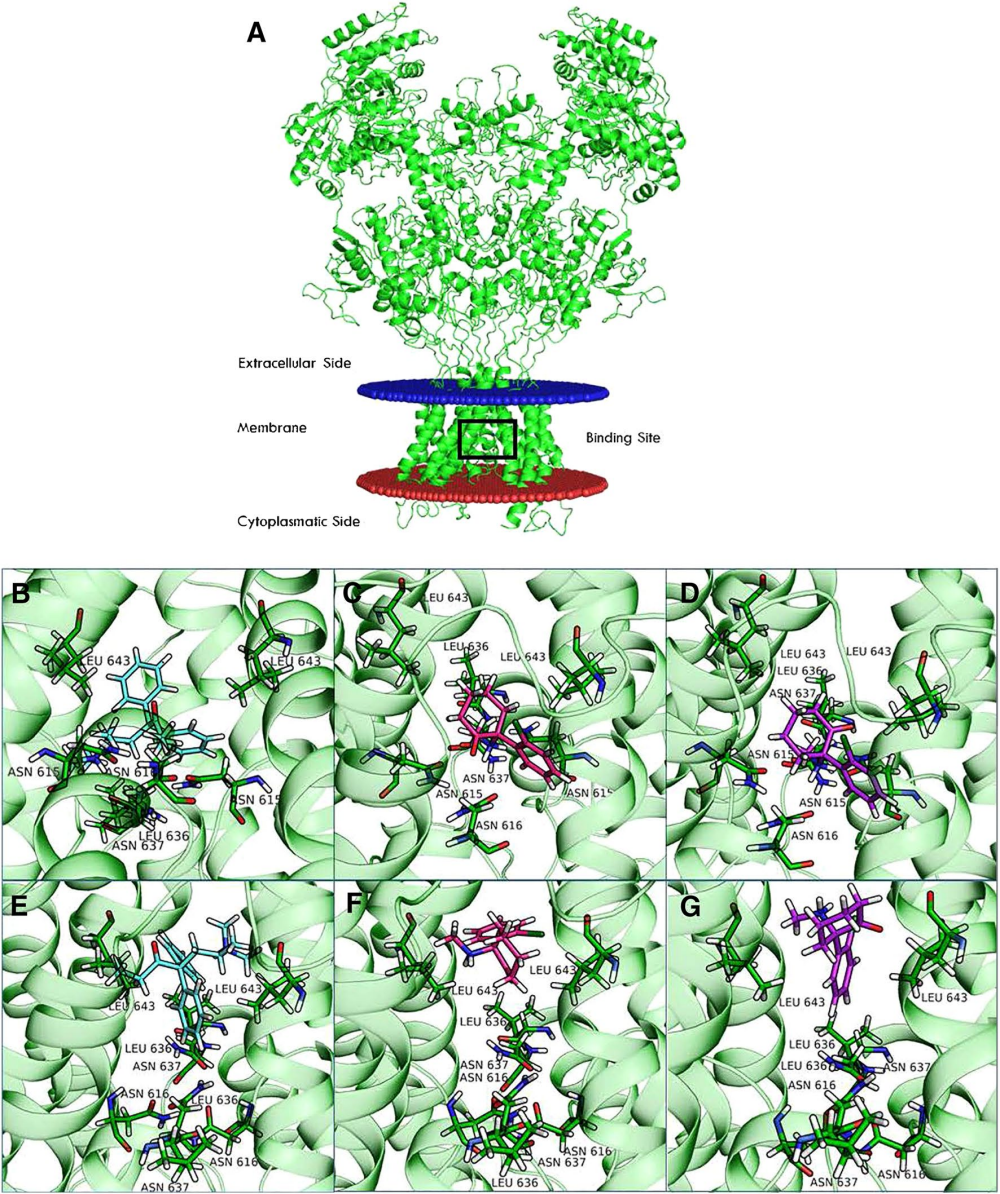
This rendering shows the interactions of uncompetitive NMDAR antagonists with the NR1-2D subtype in silico [28]. The structure of NR1-2D was obtained by electron microscopy (panel **A**, Protein Data Bank [PDB] code 6WHT). The black box highlights the drug-binding site. Structures of the complexes between esmethadone (light blue), arketamine (magenta), and esketamine (purple) with NR1-2D in the open conformation model (PDB code 6WHT) and the closed conformation model (PDB code 6WHS) can be seen in panels (**B–D**) and (**E–G**) [28]

The interactions of esmethadone with the NMDAR have been recently characterized in silico (Fig. 2) [28]. The in vitro activity of esmethadone has been compared with other uncompetitive NMDAR antagonists (Tables 1, 2 and 3) [28]. Furthermore, the known influence of physiological magnesium on NMDAR subtype preference by uncompetitive NMDAR antagonists [29] has also been characterized for esmethadone (Table 4) [28].

**Table 1:** In vitro and in vivo preclinical studies with esmethadone

Author (year) and study title	Objective	Results
Bettini et al. (2022) Pharmacological comparative characterization of REL-1017 (esmethadone-HCl) and other NMDAR channel blockers in human heterodimeric N-methyl-D-aspartate receptors	To characterize REL-1017 (esmethadone-HCl) and NMDARs in silico and together with dextromethorphan, memantine, (±)-ketamine, and MK-801 in cell lines over-expressing NMDAR subtypes using a fluorometric imaging plate reader, automated patch-clamp, and manual patch-clamp electrophysiology	The pharmacological characteristics of REL-1017 at NMDARs included relatively low affinity at the NMDAR, NR1-2D subtype preference in the presence of 1 mM Mg^2+^, trapping similar to (±)-ketamine, and preferential docking and undocking of the open NMDAR
Bettini et al. (2022) The N-methyl-D-aspartate receptor blocker REL-1017 (esmethadone) reduces calcium influx induced by glutamate, quinolinic acid, and gentamicin	To study the effects of quinolinic acid and gentamicin, with or without L-glutamate and REL-1017, on intracellular calcium ([Ca^2+^]_in_) influx using the fluorometric imaging plate reader assays in recombinant cell lines expressing human GluN1-GluN2A, GluN1-GluN2B, GluN1-GluN2C, and GluN1-GluN2D NMDAR subtypes	REL-1017 reduced [Ca^2+^]_in_ induced by L-glutamate alone and when increased by quinolinic acid and gentamicin. REL-1017 may protect cells from excessive calcium entry via NMDARs hyperactivated by endogenous and exogenous molecules
Hanania et al. (2020) The N-methyl-D-aspartate receptor antagonist d-methadone acutely improves depressive-like behavior in the forced swim test performance of rats	To compare esmethadone and ketamine in the forced swim test 24 h following a single-dose administration of these two drugs in Sprague–Dawley rats	Tested doses of esmethadone (10, 20 and 40 mg/kg s.c.) and ketamine (10 mg/kg i.p.) significantly (p < 0.05) decreased the time of immobility compared with vehicle
		The reduction of immobility with 20 and 40 mg/kg of d-methadone was greater than that obtained with 10 mg/kg ketamine
Fogaça et al. (2019) N-methyl-D-aspartate receptor antagonist d-methadone produces rapid, mTORC1-dependent antidepressant effects	To examine the antidepressant action of esmethadone via mTORC1 signaling and synaptic changes in the medial prefrontal cortex of Sprague–Dawley rats	Esmethadone 20 mg/kg s.c. and ketamine 10 mg/kg i.p. increased synaptic proteins and enhanced synaptic function in the medial prefrontal cortex
		REL-1017 induces rapid antidepressant-like actions through BDNF-dependent synaptic plasticity in the medial prefrontal cortex
Henningfield et al. (2022) REL-1017 (esmethadone; d-methadone) does not cause reinforcing effect, physical dependence and withdrawal signs in Sprague Dawley rats	Two studies were performed to evaluate lack of reinforcing effect, physical dependence, and withdrawal of REL-1017 in Sprague–Dawley rats (1) Self-administration Study: rats were trained to self-administer oxycodone intravenously (IV) and then were subjected to 3-day substitution tests where saline, oxycodone, and REL-1017 were self-delivered IV by a fixed number of lever presses (2) Drug Discontinuation Study: rats were treated for 30 days by oral gavage with vehicle, REL-1017, ketamine, or morphine and evaluated for withdrawal with functional observational batteries	In the self-administration study, saline, vehicle, and all doses of esmethadone showed the typical “extinction burst” pattern of response, characterized by an initial rapid increase of lever-pressing followed by decrease over 3 days. Rats treated with oxycodone maintained stable self-injection, as expected for reinforcing stimuli
		In the withdrawal study, esmethadone did not engender either morphine or ketamine withdrawal signs over 9 days following abrupt discontinuation of drug exposure
		REL-1017 showed no evidence of abuse potential and did not engender withdrawal symptomatology
Bifari et al. (2022) REL-1017 (esmethadone), a novel NMDAR blocker for the treatment of MDD is not neurotoxic in Sprague–Dawley rats	To determine in Sprague–Dawley rats if the administration of esmethadone via oral gavage for 1–4 days could produce Olney’s lesions and cortical neuronal death and microgliosis as compared with MK-801, a known potent neurotoxic NMDAR blocker	Administration of esmethadone at low (20–31.25 mg/kg in females and males), medium (40–62.5 mg/kg), or high (80–110 mg/kg) doses did not cause pathomorphological changes in brain neurons and did not impair behavior and activity

**Table 2:** IC_50_ values of esmethadone and reference NMDAR blockers

	NR1-2A			NR1-2B			NR1-2C			NR1-2D		
	IC_50_ (µM)	Slope	Min. (%)	IC_50_ (µM)	Slope	Min. (%)	IC_50_ (µM)	Slope	Min. (%)	IC_50_ (µM)	Slope	Min. (%)
Esmethadone	43	− 1.0	30	25	− 1.1	14	23	− 0.84	15	68	− 0.68	47
(±)-Ketamine	30	− 0.76	23	6.3	− 0.78	8	3.4	− 0.83	8	11	− 1.1	12
Memantine	34	− 0.82	29	10	− 0.86	11	3.6	− 0.82	13	7.3	− 0.88	18
Dextromethorphan	51	− 0.80	35	15	− 0.89	14	5.2	− 1.0	14	28	− 1.2	43
MK-801	0.29	− 0.69	4	0.07	− 0.94	4	0.58	− 1.0	7	0.76	− 1.2	11

IC_50_ values of five selected NMDAR channel blockers were obtained via fluorometric imaging plate reader (FLIPR) assay [28]. Fitting values were obtained for every heterodimeric NMDAR via logistic equation in GraphPad Prism v8.0. Slope is also reported in the table, as well as the minimal % Ca^2+^ influx measured in the presence of 100 µM blocker, the highest tested concentration. For example, 100 µM esmethadone reduced Ca^2+^ influx elicited by 10 µM L-glutamate by 15% in the 2C-containing cell line

**Table 3:** K_B_ and affinity ratio values of esmethadone and reference NMDAR blockers

	NR1-2A		NR1-2B		NR1-2C		NR1-2D	
	K_B_ (µM)	Affinity ratio (%)	K_B_ (µM)	Affinity ratio (%)	K_B_ (µM)	Affinity ratio (%)	K_B_ (µM)	Affinity ratio (%)
Esmethadone	8.9	51	6.1	74	4.5	100	7.8	58
(±)-Ketamine	4.3	11	1.1	42	0.46	100	1.4	33
Memantine	3.6	8	0.58	48	0.28	100	0.59	47
Dextromethorphan	9.6	13	1.9	63	1.2	100	6.7	18
MK-801	0.11	44	0.048	100	0.14	34	0.15	32

Estimated K_B_ values for five NMDAR channel blockers were obtained via FLIPR assay by L-glutamate concentration–response curves. An operational equation for allosteric modulators was used to estimate K_B_ and % affinity ratio for all tested molecules [28]

**Table 4:** IC_50_ values of esmethadone in presence of Mg^2+^

	Esmethadone IC_50_ in 1 mM MgCl_2_	Hill slope	Cell number
NR1-2A	63.1	1.06	2–8
NR1-2B	41.7	1.17	2–7
NR1-2C	28.4	1.49	2–8
NR1-2D	13.5	1.42	3–7

Experiments were conducted in whole-cell patch-clamp electrophysiology at a holding potential of – 60 mV. Esmethadone concentration–response curves were obtained via whole-cell manual patch-clamp recordings in the presence of sub-saturating 1 µM L-glutamate, 10 µM glycine, and 1 mM MgCl_2_. Every clamped cell was assessed with a single concentration of esmethadone, and the cell number range indicates the minimum and the maximum number of clamped cells per concentration for each NMDAR subunit-expressing cell type. Esmethadone was found to be approximately fivefold more potent in blocking NR1-2D subtypes compared to NR1-2A subtypes. Fittings parameters for esmethadone were obtained from data shown in [28] and analyzed with GraphPad Prism v8.0

The pharmacological interactions of esmethadone with human heterodimeric NMDARs described by Bettini and colleagues highlighted low NMDAR receptor affinity, NR1-2D subtype preference, ketamine-like trapping in the channel pore, and a propensity for undocking from the NMDAR in the open conformation. Importantly, the unique characteristics of esmethadone’s interaction with NMDARs, along with its lower potency compared to ketamine [28], may explain the lack of dissociative effects seen in clinical trials [25, 30]. Similarly, the ketamine enantiomer arketamine may be effective as an antidepressant with fewer dissociative effects because of its lower NMDAR affinity as compared to the ketamine enantiomer esketamine [31]. Other NMDAR antagonists, such as memantine and lanicemine, may lack consistent antidepressant effects in patients with MDD because of their low trapping [32] as compared to the higher trapping shown by ketamine and esmethadone. Additional in vitro experiments showed that esmethadone reduces Ca^2+^ influx induced by L-glutamate at very low concentrations, as well as Ca^2+^ influx due to quinolinic acid (QA) and gentamicin stimulation. Therefore, esmethadone may protect cells from the excessive calcium entry via NMDARs that are hyperactivated by very low concentrations of glutamate and by endogenous (e.g., QA) and exogenous (e.g., gentamicin) molecules [33].

Two clinical studies designed to assess the human abuse potential and performed in recreational drug users showed no meaningful abuse potential for esmethadone in this patient population [34, 35]. In these studies, dextromethadone was compared to oxycodone, ketamine, and dextromethorphan. Dextromethorphan is an over-the-counter antitussive drug and NMDAR uncompetitive antagonist with affinity for the NMDAR that is approximately threefold higher than esmethadone [28]. The primary metabolite of dextromethorphan, dextrorphan, also has NMDAR affinity [36], in contrast with 2-ethylidene-1,5-dimethyl-3,3-diphenylpyrrolidine (EDDP), the primary metabolite of esmethadone, which is inactive. These differences in potency and metabolism may explain the higher drug liking score of 300 mg oral dextromethorphan compared to 150 mg oral esmethadone in recreational drug users, as reported by Shram and colleagues [34].

Notably, cortical neurons of rats exposed to high doses of esmethadone did not show evidence of Olney’s lesions or other neuropathological changes [37], in contrast with other uncompetitive NMDAR antagonists known to produce Olney’s lesions [38–41]. This lack of evidence for potential neurotoxicity may be related to the relatively lower affinity of esmethadone binding at NMDARs, as demonstrated in radioligand binding assays, fluorometric imaging plate reader assays, and automated and manual patch assays [27, 28].

### Lack of opioid activity by esmethadone: in vitro, animal, and human evidence

Since the introduction of methadone in the US in 1946 [42] and because of the structural similarity with levomethadone (the opioid-active mu agonist levo-enantiomer), many studies have examined the interactions of the dextro-enantiomer esmethadone with opioid receptors and its potential for eliciting opioid agonist effects in animal models and humans. Receptor affinity studies using esmethadone in rat models show 20-fold lower affinity for mu opiate receptors compared to the opioid-active enantiomer, levomethadone [43]. We performed two radioligand binding assays at human opioid receptors using esmethadone, levomethadone, and EDDP (Relmada studies performed by Eurofins: TW04-0009163 and TW04-0009695, submitted to FDA under IND 133345). In these studies, esmethadone exhibited a 27- to 40-fold lower affinity for human mu opioid receptors as compared to levomethadone (IC_50_ 610/410 nM and IC_50_ 14.6/14.7 nM for esmethadone and levomethadone, respectively). The major metabolite of esmethadone, EDDP, had no meaningful opioid affinity (Relmada studies submitted to FDA under IND 133345).

Animal studies show a lack of meaningful opioid effects and lack signs of withdrawal after abrupt discontinuation of esmethadone [44–46]. Furthermore, the results of these earlier preclinical studies were replicated in recent studies that showed esmethadone does not cause reinforcing effects, physical dependence, or withdrawal in rats [47]. These preclinical studies are corroborated by early human studies indicating no meaningful abuse potential from esmethadone [42, 48, 49] and by more recent clinical studies employing state-of-the-art methodology [34, 35, 47]. Taken together, preclinical and clinical studies confirm this 2019 Drug Enforcement Administration statement: “The d-isomer lacks significant respiratory depressant action and addiction liability, but possesses antitussive activity” [50]. The lack of opioid activity of esmethadone, in contrast with the opioid activity of levomethadone, is in line with the known stereoselectivity of opioid agonist activity for opioid enantiomers: esmethadone, dextromethorphan, and dextro-morphine are all inactive at opioid receptors, in contrast with the opioid agonist drugs levomethadone, levomethorphan, and levo-morphine [42, 43, 45, 51, 52]. Finally, the successful substitution of racemic methadone with half the dose of levomethadone in over 1500 patients with opioid use disorder indirectly supports the lack of opioid activity of esmethadone [53].

While the scientific evidence for esmethadone’s lack of meaningful opioid agonist activity is conclusive, the layman’s assumption may still be one of similarity of opioid effects to racemic methadone and levomethadone. This erroneous assumption may need additional educational efforts from the scientific community and from treating physicians to dispel addiction concerns that are unsupported by scientific data and that may interfere with its potential use as an antidepressant.

### Antidepressant-like activity of esmethadone: preclinical studies

Esmethadone has rapid antidepressant-like activity in the rat forced swim test [54], an established model of depressive-like behavior predictive of antidepressant effects in humans. Aside from reversing depressive-like behavior in preclinical paradigms of depression, esmethadone, similarly to ketamine, may also reverse neuronal dysfunctions associated with depressive-like behavior by increasing synaptic spine volume and restoring spinogenesis [14, 16]. Remarkably, the reversal of depressive-like behavior by esmethadone and other NMDAR antagonists appears to rely on the restoration of synaptic proteins via a BDNF-dependent mechanism [14, 15, 55]. Figure 1 shows a current molecular hypothesis for the rapid relief of depressive behaviors and associated symptoms by esmethadone and other uncompetitive NMDAR antagonists [17]. While NMDAR antagonism is thought to be the mechanism of action of the antidepressant effects of uncompetitive NMDAR channel blockers, activity at other receptor systems, including opioid receptors [19] and sigma receptors [20], is also hypothesized. Esmethadone, aside from its uncompetitive NMDAR antagonist activity, shows affinity for other receptors (Table 5), which may also be implicated in its potential therapeutic effects.

**Table 5:** Esmethadone affinities for NMDARs and additional binding sites

Target	Concentration	% Inhibition	Species
Calcium channel L-type, benzothiazepine	10 µM	81	Rat
Calcium channel L-type, phenylalkylamine	10 µM	81	Rat
Glutamate, NMDA, phencyclidine	10 µM	73	Rat
Histamine H_1_	10 µM	72	Human
Muscarinic M_5_	10 µM	72	Human
Muscarinic, oxotremorine-M	10 µM	52	Rat
µ-Opioid receptor (PO3, MOP)	10 µM	90	Human
Serotonin (5-hydroxytryptamine) 5-HT_2C_	10 µM	89	Human
Serotonin (5-hydroxytryptamine) 5-HT_5A_	10 µM	70	Human
Serotonin (5-hydroxytryptamine) 5-HT_7_	10 µM	66	Human
Sigma σ1	10 µM	85	Human
Sodium channel, site 2	10 µM	69	Rat
Serotonin transporter (5-hydroxytryptamine)	10 µM	73	Human (SERT)

The activity of esmethadone was determined in radioligand binding assays through Eurofins Discovery Services (Relmada data on file). Results are presented as the percent inhibition of specific binding activity. Values listed above met criteria for significance (≥ 50% inhibition or stimulation)

### Clinical studies assessing safety, tolerability, and efficacy of esmethadone in MDD

The safety, tolerability, and efficacy of esmethadone was assessed in two phase 1 trials and one phase 2 trial (Table 6). A single ascending dose (SAD) clinical trial demonstrated safety and tolerability of esmethadone in single doses of up to 150 mg. The 150 mg dose was deemed the maximum tolerated dose (MTD) based on the insurgence of nausea and vomiting. No patient experienced opioid-like euphoria or ketamine-like dissociative symptoms [30]. The lack of esmethadone-induced opioid-like euphoria and lack of ketamine-like dissociation at MTD was also confirmed in two studies designed to assess human abuse potential [34, 35]. The safety and tolerability of esmethadone administered daily at doses of 25 mg, 50 mg, and 75 mg for 10 days were then tested in a multiple ascending dose (MAD) trial [30]. In these subjects, there was no evidence of withdrawal after abrupt discontinuation of the 10-day course of esmethadone.

**Table 6:** Human safety and efficacy: phase 1 and phase 2 studies with esmethadone

Author (year) and study title	Study design		Sample size (age group)	Treatment groups and duration	Objective	Results
Bernstein et al. (2019) Characterization of the safety and pharmacokinetic profile of D-methadone, a novel N-methyl-D-aspartate receptor antagonist in healthy, opioid-naïve subjects	Two phase 1, double-blind, randomized, placebo-controlled single ascending dose (SAD) and multiple ascending dose (MAD) studies	Phase 1 SAD	42 healthy subjects (18–55 years)	A total of 31 subjects received esmethadone, and a total of 11 subjects received placebo	Safety and tolerability of esmethadone compared to placebo	Single doses of 5 mg, 20 mg, 60 mg, 100 mg, and 150 mg of esmethadone and daily doses up to 75 mg for 10 days were well tolerated, with mostly mild treatment-emergent adverse events and no severe or serious adverse events
				In each cohort (5 mg, 20 mg, 60 mg, 100 mg, 150 mg), eight subjects were randomly assigned to receive placebo (*n* = 2) or esmethadone (*n* = 6), except for 200 mg cohort (placebo *n* = 1; REL-1017 *n* = 1)		
				Single oral dose		
		Phase 1 MAD	24 healthy subjects (18–55 years)	A total of 18 subjects received esmethadone, and a total of six subjects received placebo	To determine pharmacokinetic parameters	The maximum tolerated dose was 150 mg due to nausea and vomiting. There were no clinically meaningful opioid or psychotomimetic effects and no QTc-related adverse events
				In each cohort (25 mg, 50 mg, 75 mg), eight subjects were randomly assigned to receive placebo (*n* = 2) or esmethadone (*n* = 6)		
				Daily oral dose for 10 days		
Fava et al. (2022) REL-1017 (esmethadone) as adjunctive treatment in patients with major depressive disorder: a phase 2a randomized double-blind trial	Phase 2, double-blind, randomized, placebo-controlled study to assess efficacy and safety of two dosages of esmethadone, 25 mg and 50 mg, in patients with MDD experiencing a major depressive episode (MDE) with inadequate response to one to three courses of antidepressant treatment		62 adults (18–65 years) with MDD experiencing a current MDE and inadequate response to one to three courses of antidepressant treatment	Twenty-two patients received placebo	Safety, tolerability, and pharmacokinetic (PK) evaluations	No psychotomimetic or opioid effects. No evidence of withdrawal. No adverse events (AEs) related to QTc prolongation
				Nineteen patients received 25 mg esmethadone (75 mg loading dose on day 1)		
				Twenty-one patients received 50 mg esmethadone (100 mg loading dose on day 1)	Efficacy outcomes (changes in MADRS, CGI-I, SDQ scores compared to placebo)	Esmethadone showed efficacy at day 4 that was sustained up to day 14. The effect size ranged from 0.7 to 1.1
				Treatment and duration: single daily oral dose for 7 days		

In these SAD and MAD studies [30], esmethadone exhibited linear pharmacokinetics with dose proportionality for most single-dose and multiple-dose parameters. Single doses up to 150 mg and daily doses up to 75 mg for 10 days were well tolerated with mostly mild treatment-emergent adverse events and no severe or serious adverse events. There was no evidence of respiratory depression, dissociative and psychotomimetic effects, or withdrawal signs and symptoms upon abrupt discontinuation. In regard to the effects of esmethadone on the QTc interval, an overall dose–response effect was observed, with higher doses resulting in larger QTcF (QT interval corrected using the Fridericia formula) changes from baseline. Importantly, none of the changes was considered clinically significant. Similar effects of the QTcF were observed in the phase 2 study [25]. No detectable conversion of esmethadone to levomethadone occurred in vivo.

Two randomized, double-blind, active- and placebo-controlled crossover studies were designed to evaluate the abuse potential of esmethadone compared with oxycodone (oxycodone study) or ketamine (ketamine study) in healthy recreational drug users. Three doses of esmethadone were evaluated in each study: 25 mg (the proposed therapeutic daily dose for MDD treatment), 75 mg (loading dose), and 150 mg (MTD). Positive controls were 40 mg oral oxycodone in the oxycodone study and 0.5 mg/kg intravenous ketamine infused over 40 min in the ketamine study. The ketamine study included 300 mg oral dextromethorphan as an exploratory comparator. The primary endpoint was the maximum effect (E_max_) for drug liking, assessed using a bipolar 100-point visual analog scale (VAS). In the oxycodone study and the ketamine study, 47 and 51 participants completed all treatment arms, respectively. In both studies, esmethadone doses ranging from therapeutic (25 mg) to six times therapeutic (150 mg) had a statistically significant and clinically meaningful (p < 0.001) lower drug liking VAS E_max_ compared with positive controls. Results were consistent for all secondary endpoints, including measurements of overall drug liking and willingness to take the drug again, in both studies. Moreover, in the ketamine study, drug liking VAS E_max_ scores for esmethadone at all tested doses were significantly lower versus dextromethorphan (p < 0.05) (exploratory endpoint). In conclusion, these studies indicated no meaningful abuse potential for esmethadone.

The safety, tolerability, and efficacy of esmethadone were tested in a phase 2 study [25]. This study aimed to examine the effects of esmethadone in patients with MDD with inadequate response to standard antidepressants during the course of a major depressive episode. This was a randomized, double-blind, placebo-controlled trial, comprising three arms, designed to assess the safety, tolerability, pharmacokinetics, and efficacy of two dosages of esmethadone (25 mg or 50 mg orally once a day) administered for 7 days and conducted in ten centers across the United States. Patients were randomly assigned in a 1:1:1 ratio to placebo (*N* = 22), 25 mg/day esmethadone (*N* = 19), or 50 mg/day esmethadone (*N* = 21). All patients were maintained on their stable dose of standard antidepressant. Safety scales included the four-item Positive Symptom Rating Scale for psychotomimetic symptoms, the Clinician-Administered Dissociative States Scale for dissociative symptoms, the Clinical Opiate Withdrawal Scale for withdrawal signs and symptoms, and the Columbia Suicide Severity Rating Scale for suicidality. Efficacy was evaluated based on changes in the Montgomery–Åsberg Depression Rating Scale (MADRS) score. All 62 randomly assigned patients were included in the full analysis set population. Patients experienced only mild to moderate transient adverse events, and there was no evidence of dissociative, psychotomimetic, or opioid effects or withdrawal signs and symptoms, confirming the safety and tolerability results of phase 1 studies [30]. Clinically meaningful and statistically significant improvement in MADRS score started on day 4 with both esmethadone doses and was sustained through day 7 (last dose) and day 14 (7 days after the last dose), with effect sizes from 0.7 to 1.0. This trial confirmed the very favorable safety, tolerability, and pharmacokinetic profiles of esmethadone and indicated that esmethadone had rapid and sustained antidepressant effects compared with placebo in patients with inadequate responses to antidepressant treatments.

Table 7 lists publications from phase 1 and phase 2 sub-analyses.

**Table 7:** Sub-analyses and case reports

Author (year) and study title	Study sub-analysis	Results
De Martin et al. (2021) REL-1017 (esmethadone) increases circulating BDNF levels in healthy subjects of a phase 1 clinical study	Sub-analysis of the phase 1 (MAD) clinical study (Bernstein et al., 2019) to assess plasma BDNF levels after 25 mg of REL-1017 orally administered for 10 days	Plasma BDNF levels were significantly higher in REL-1017-treated subjects compared to the placebo group
		The increase started on day 2 and was maintained throughout day 10
Guidetti et al. (2022) Sub-analysis of subjective cognitive measures from a phase 2, double-blind, randomized trial of REL-1017 in patients with major depressive disorder	Sub-analysis of subjective cognitive measures from a phase 2, double-blind, randomized trial of REL-1017 (Fava et al. [25])	Esmethadone significantly improved subjective measures of cognitive impairment, in addition to improving total MADRS and SDQ scores
Guidetti et al. (2022) REL-1017 (esmethadone) may rapidly reduce dissociative symptoms in adults with major depressive disorder unresponsive to standard antidepressants: a report of 2 cases	Sub-analysis of Clinician-Administered Dissociative States Scale (CADSS) scores from the phase 2, double-blind, randomized trial of esmethadone (Fava et al. [25])	There were two patients with elevated baseline CADSS scores. Both patients had meaningful CADSS score improvement on day 1 (2 h after the first dose of esmethadone). The improvement was sustained on day 7 (2 h post-dose) and 2 days after treatment discontinuation, on day 9, with complete resolution of dissociative symptoms (CADSS total score of 0)

## Uncompetitive NMDAR antagonists: pharmacokinetics, tolerability, and safety considerations

Among the upcoming pharmacological class of NMDAR antagonists that may work as rapid antidepressants in patients, esmethadone stands out because of its very favorable tolerability and safety profile. The efficacy and safety of esmethadone may be determined by its selectivity for tonically hyperactive NR1-2D subtypes at doses therapeutic for MDD [28]. In addition, esmethadone has an ideal pharmacokinetic profile that allows once-daily oral administration [25, 30]. Ketamine and its enantiomers can only be administered intravenously or intranasally due to variable oral absorption. In addition, the safety window for ketamine and esketamine may be too narrow: at dosages in current use for the treatment of depression, approximately 70% of patients experience dissociative symptoms [56]. The combination drug dextromethorphan–bupropion is better tolerated than ketamine and esketamine [22] but carries the combined side effects of two different drugs with the burdens of polypharmacy, which may be especially relevant when this combination drug is under consideration for patients who are already taking other drugs.

Furthermore, ketamine and dextromethorphan have been reported to cause Olney’s lesions in rats. While the significance of this neuropathological animal finding is unknown, it cannot be discounted. Up to recently, the therapeutic uses of ketamine (for anesthesia) and dextromethorphan (for cough suppression) have been intermittent. Their current use for the treatment of MDD is likely to be chronic. The safety of the chronic uses of ketamine and dextromethorphan will need to be confirmed in post-marketing analyses. In contrast, esmethadone does not cause Olney’s lesions in rats [33], suggesting that its long-term use may be safer compared to NMDAR antagonists that have been found to cause these lesions. The safety of esmethadone is also indirectly supported by over 70 years of chronic racemic methadone use in millions of patients with pain and opioid use disorder. Most of these patients are exposed to esmethadone serum levels greater than those seen in patients treated with the dose proposed for MDD. The average methadone dose for opioid use disorder is approximately 75 mg daily, and 50% of this dose is esmethadone. The esmethadone exposure in these patients with opioid use disorder and pain is, therefore, higher than the exposure of patients with MDD treated with 25 mg esmethadone. No long-term detrimental neurological consequences have been described in patients treated chronically with racemic methadone.

In conclusion, due to the favorable pharmacological features described above, if ongoing phase 3 studies confirm the promising phase 2 results, esmethadone may potentially become the best-in-class agent for safety, tolerability, and efficacy among uncompetitive NMDAR antagonists with rapid antidepressant effects.

## Data Availability

Not applicable.
